# Is your syringe services program cost-saving to society? A methodological case study

**DOI:** 10.1186/s12954-021-00575-4

**Published:** 2021-12-07

**Authors:** Don C. Des Jarlais, Jonathan Feelemyer, Courtney McKnight, Kelly Knudtson, Sara N. Glick

**Affiliations:** 1 Epidemiology, Social and Behavioral Sciences, School of Global Public Health, New York University, 708 Broadway, 7th Floor, New York, NY 10003 USA; 2 Division of Allergy and Infectious Diseases, Department of Medicine, University of Washington, Seattle, WA USA

**Keywords:** Syringe service programs, Persons who inject drugs, HIV, Cost-saving analysis

## Abstract

**Background:**

While there is a general acceptance among public health officials and policy-makers that syringe services programs can be effective in reducing HIV transmission among persons who inject drugs, local syringe services programs are often asked to provide economic justifications for their activities. A cost-effectiveness study, estimating the cost of preventing one HIV infection, would be the preferred methods for addressing this economic question, but few local syringe services programs have the needed data, staff and epidemiologic modeling resources needed for a cost–effectiveness study. We present a method for estimating a threshold value for the number of HIV infections prevented above which the program will be cost-saving to society. An intervention is considered “cost-saving” when it leads to a desirable health outcome a lower cost than the alternative.

**Methods:**

The research literature on the effectiveness of syringe services programs in controlling HIV transmission among persons who inject drugs and guidelines for syringe services program that are “functioning very well” were used to estimate the cost-saving threshold at which a syringe services program becomes cost-saving through preventing HIV infections versus lifetime treatment of HIV. Three steps are involved: (1) determining if HIV transmission in the local persons who inject drugs (PWID) population is being controlled, (2) determining if the local syringe services program is functioning very well, and then (3) dividing the annual budget of the syringe services program by the lifetime cost of treating a single HIV infection.

**Results:**

A syringe services program in an area with controlled HIV transmission (with HIV incidence of 1/100 person-years or less), functioning very well (with high syringe coverage, linkages to other services, and monitoring the local drug use situation), and an annual budget of $500,000 would need to prevent only 3 new HIV infections per year to be cost-saving.

**Conclusions:**

Given the high costs of treating HIV infections, syringe services programs that are operating according to very good practices (“functioning very well”) and in communities in which HIV transmission is being controlled among persons who inject drugs, will almost certainly be cost-saving to society.

## Introduction

That syringe services programs (SSP) reduce HIV transmission among persons who inject drugs (PWID) is a conclusion supported by the overwhelming weight of the scientific evidence [1]. While there is agreement among scientists and many policy-makers about the effectiveness of SSPs, some SSPs continue to have to defend their operations. SSPs are often asked about the financial impact of their SSP activities for the local community. The ideal answer to this question would be: “We have done a cost-effectiveness study, and estimate that we are preventing *X* number of new HIV infections at a cost of *Y* dollars per infection prevented.” Very few SSPs, however, have the resources needed to conduct such a cost-effectiveness study. Citing research conducted with SSPs in other geographic areas will often be of little value in answering the question, especially if the other areas are notably different from the local area and if the studies involve complicated calculations with data that are not locally available.

Here, we outline procedures and analyses so that an SSP should be able to answer by stating: “We have analyzed the data on HIV transmission among PWID in our area and on the operations of our program, and, considering the high cost of treating HIV infections, we can be quite confident that our SSP is saving money for society.”

### Distinction between cost-effectiveness and cost-savings analyses

It will be useful to start by distinguishing between “cost-effectiveness” and “cost-savings” analyses in health economics research. The two terms are often used interchangeably but do have different technical meanings. Cost-effectiveness analyses refer to economic analyses that investigate the costs of achieving a specific health objective through a specific program. Cost-effectiveness studies usually involve detailed information on the costs of programs and complex models of the number of specific desirable health outcomes created by the program [2]. The most common desirable health objective in a cost-effectiveness analysis of an SSP would be an HIV infection that is averted through the activities of the SSP. The cost-effectiveness analysis would thus require information about the costs of the program and a detailed epidemiological model of the increased number of new HIV infections that would occur in the absence of the SSP. Developing a model of how the activities of a local SSP—educating PWID about HIV transmission, distributing sterile syringes and condoms, testing for HIV and referring HIV seropositive persons to antiretroviral therapy (ART), and persons with substance use disorders to substance use treatment—avert a specific (always estimated) number of HIV infections requires a large amount of data. This would include not only program activities but also HIV risk behaviors and HIV transmission dynamics within the local PWID population. Thus, very few SSPs will have the resources needed for a cost-effectiveness study of their program.

In a cost-savings analysis, a different question is asked: given two methods for achieving the same desirable health outcome, which method costs less? For the cost-savings analysis of an SSP presented below, the desirable health outcome is averting a death from HIV. The two methods of achieving this desirable outcome are (1) preventing the HIV infection, and (2) treating people living with HIV so that they do not die from HIV. In this report, we propose procedures for determining a lower threshold for the number of new HIV infections averted per year for a program to be cost-saving to society as compared to the discounted price of lifelong treatment for HIV. If the number of infections averted is higher than this threshold, then investing in the program would be cost-saving.

In the “Discussion” section, we also consider how such cost-savings analyses might be used in support of SSPs in policy decisions.

## Methods

### Developing a rationale that a local SSP is extremely likely to be cost-saving

Here, we present a straightforward method of determining whether a local program is highly likely to be “cost-saving,” that is whether the costs of the local program save money for society compared to the cost of treating people with HIV. The method begins with assessing whether the local SSP is likely to be effective in averting HIV infections among PWID.

### Data needs

A moderate amount of data are needed for the cost-savings analysis. These data include:

- A rough estimate of the size of the local PWID population. This need not be precise; an estimate that is correct to the order of magnitude is likely to be sufficient. An estimate based on averaging estimates of local experts—health department staff, substance use treatment staff, law enforcement and drug overdose records—is likely to be sufficient. Note, however, that many of these estimates are based on PWID who became known to these agencies over long periods of time. Thus, it may be necessary to adjust these estimates for persons who are not currently injecting and would not be in need of sterile injection equipment. This may be half of the persons who were used in making the estimate. (There have been several studies published on estimating PWID populations in US locations [3–5] if programs have the resources to conduct such studies).
- Information on program operations, particularly the numbers of syringes distributed per year and whether program participants are linked to ART treatment and substance use disorder treatment [6].
- Estimated HIV incidence and/or trends in prevalence in the local PWID population. Again, a high level of precision is not required, but it is necessary to determine if HIV incidence and prevalence are stable/decreasing versus increasing in the local PWID population. If there is a reasonable amount of HIV testing in the community, then newly diagnosed cases of HIV among persons with injecting drug use as the transmission risk can be used to assess whether HIV incidence and prevalence are likely to be stable or decreasing versus increasing. These data are available for most localities in the USA, particularly where an SSP works collaboratively with state and local health departments [7].

### Is HIV transmission is being controlled in the local PWID population?

It is difficult to argue that a local SSP is either effective or cost-saving if HIV transmission is not being controlled in the local PWID population. Indeed, if HIV transmission is not being controlled in the local PWID population, then it is highly likely that the SSP needs to be expanded and/or improved.

One issue in assessing whether HIV transmission is being controlled is that, to our knowledge, there are no HIV prevention programs that have succeeded in completely eliminating HIV transmission in any known PWID population. Thus, the total absence of new HIV infections among PWID cannot be used as a measure of controlling HIV transmission. We have previously defined an HIV incidence of 0.05/100 person-years at risk as “ending an HIV epidemic” in a PWID population and would consider a stable incidence rate of approximately 1/100 person-years as evidence that HIV transmission is being controlled in a PWID population [8].

Directly measuring low HIV incidence rates among PWID can be both logistically complicated and quite expensive. If HIV testing is widespread in the local PWID population, then trends in HIV seroprevalence among PWID and trends in newly identified cases of HIV infection among PWID can be used as indirect measures of HIV incidence [9]. If HIV prevalence is low (less than 10%) and stable or declining, then this would be an indication that HIV incidence is also low. If the numbers of newly identified cases of HIV infection among PWID are low (approximately 1% per year of the estimated HIV-negative PWID population) and the numbers of newly identified cases or HIV infection are stable or declining, this would also be an indication that HIV transmission is being controlled. If HIV prevalence is rising or the number of newly identified cases of HIV is increasing, then immediate efforts to address the situation are needed. Conclusions about control of HIV transmission should not be made until stability has been established.

### The SSP program is “functioning very well”

Very low HIV incidence among PWID is not by itself sufficient evidence that SSPs and other HIV prevention programs are effective and cost-saving. The low incidence may be the result of the absence of a sufficiently large introduction of HIV into the local PWID population [10]. Making a case that the local SSP is effective and is cost-saving requires considering whether the program is “functioning very well.” We now have over 35 years of experience with and research on SSPs, during which a number of good operational practices have been identified.

#### High syringe coverage/large numbers of syringe distributed

Controlling HIV and HCV transmission requires access to very large numbers of sterile syringes for PWID to prevent multi-person use (sharing) of syringes. The Centers for Disease Control and Prevention (CDC) recommends that PWID use a new syringe for each injection [11]. The Joint United Nations Programme on HIV/AIDS (UNAIDS) has set current targets for needle/syringe programs. Distributing up to 100 new syringes per PWID per year is considered “low” coverage, and from 100 to 200 new syringes per PWID per year is “medium” coverage, and 200 or more new syringes per PWID per year is “high” coverage [12]. Achieving the CDC goal or UNAIDS target of “high coverage” may not be fully necessary for controlling HIV transmission in a PWID population, but the recommendations do reflect a consensus in the field that PWID need access to large numbers of sterile syringes.

The simplest method of assessing whether an SSP is distributing sufficient amounts of sterile injection equipment may be simply talking with PWID—those coming to the program, PWID entering substance use treatment, and PWID in the community. When asked, do PWID reply that they, individually, and that their peers in the community have sufficient supplies of sterile syringes to avoid having to share? If a very large percentage of PWID reply yes for both themselves and their peers, then the SSP is probably distributing sufficient supplies. If a substantial minority—perhaps 10% or more—of PWID say that either they personally or that their peers are not obtaining sufficient supplies, then the program should explore methods for increasing the supply of sterile injection equipment.

#### Linking participants to substance use treatment and HIV treatment

Highly effective SSPs do not operate in isolation but as part of community systems of “combined prevention and care” for PWID [13]. SSP participants who are living with HIV should be linked to ART. ART serves to both increase the health and well-being of the patients, and also reduces infectiousness and thus lowers transmission risk to others.

SSPs should also be linking participants with problematic substance use to substance use treatment programs. It is critical that such linkage be voluntary for the participants, and not in any way coercive [14–16]. Medication for opioid use disorders (MOUD)—methadone and buprenorphine in particular—have been shown to greatly reduce injecting drug use and to improve the health and social functioning of PWID [17–19]. The reductions in drug injection reduce the likelihood that HIV seronegative PWID will acquire HIV and the likelihood that HIV seropositive PWID will transmit the virus to others [20, 21].

#### Monitoring the local drug use situation through information obtained from program participants and other stakeholders

Local drug use situations can change rapidly, and such changes may create increased risk for HIV transmission. The introduction of cocaine injecting, which can greatly increase the frequency of injecting and require much greater supplies of sterile syringes, has served as a dramatic example [22]. Monitoring the local drug use situation can be done with informal discussions, qualitative interviews, focus groups and formal structured surveys, which may include HIV and HCV testing. As noted above, monitoring the local drug use situation can also provide critical information regarding the syringe coverage in the local area. If the monitoring should detect a situation that threatens increased HIV transmission, the SSP should immediately explore the situation in greater depth and adjust services to minimize any possible outbreak of HIV transmission.

### “Functioning very well” versus “operating according to best practices.”

We considered using the term “operating according to best practices” instead of the term “functioning very well” for assessing the functioning of an SSP. Clearly “operating according to best practices” would be a higher and preferable standard than “functioning very well.” However, many SSPs have to operate under conditions that preclude “best practices.” Examples would include programs that are legally required to do “1:1” exchange and programs that operate in areas without sufficient substance use treatment programs to which PWID in need of treatment could be referred. We do believe that SSPs can be powerful interventions, and that even with modest restrictions, they can still be very effective in controlling HIV transmission among PWID and be cost-saving. Thus, we chose the term “functioning very well” to permit some flexibility in assessing SSP operations rather than the absolutist term of “operating according to best practices.”

Demonstrating that a local SSP is “functioning very well” does not guarantee that the program is either effective at preventing community transmission of HIV or is cost-saving for society. However, if a program is not “functioning very well” according to the three standards noted above, it is unlikely that it is preventing community transmission of HIV to the degree that it should.

### Calculating whether a local SSP is likely to be cost-saving

If the local SSP is functioning according to the three practices described above, and if available evidence indicates that HIV transmission is being controlled in the local PWID population, addressing the question of whether the local SSP is likely to be cost-saving to the society can be done through relatively simple calculations.

A 2015 study estimated the discounted cost of avoiding one HIV infection at $229,899. (This cost is discounted to present value in 2015 dollars [23]). Dividing the annual budget of the SSP by $229,899 and rounding up to the nearest whole number gives the minimum number of infections to be prevented in order to be cost-saving to society.

## Example results

Table 1 provides an illustrative example of applying our methods to assess whether a hypothetical SSP is cost saving to society.

**Table 1:** Illustrative example for assessing whether a local SSP is cost-saving to society

Size of the local PWID population
There has not been any formal study to estimate the size of the local PWID population. Estimates for local experts—the Health Department, substance use treatment staff, local hospital staff, law enforcement, and the SSP staff themselves range from 3000 to 7000, with an average estimate of 5000 PWID active an any point in time
Is HIV transmission among PWID under control in the local area?
HIV testing is readily available in the area
The SSP, the substance use treatment programs, and the local health department all offer no cost HIV testing. The health department does conduct HIV surveillance based on the widespread availability of testing
The number of newly identified cases of HIV infection among persons with injecting drug use as their transmission risk (a surrogate measure of incidence) has remained stable at 50 ± 10 per year over the last 5 years. The number of PWID living with HIV (a proxy measure of HIV prevalence) is approximately 500 and has been growing slightly, as there are relatively few deaths among PWID infected with HIV. (This measure can be estimated by subtracting known deaths among PWID infected with HIV from the total of PWID diagnosed with HIV over time.)
Conclusion: HIV transmission among PWID is under control in the area
Is the SSP “functioning very well?
The SSP distributes about 500,000 syringes per years. It works on a non-strict 1 for 1 model and encourages secondary exchange
Persons obtaining large numbers of syringes for secondary exchange are required to bring in large numbers of used syringes
The SSP program provides “starter kits” so that all participants leave the exchange with some sterile syringes even if they did not have any used syringes to bring to the exchange
Some pharmacies in the area also sell syringes to persons who inject drugs
Informal interviews with SSP participants, persons entering substance abuse treatment, and PWID in the community indicate that PWID believe they have very good access to sterile syringes, and that sharing because of a lack of sterile syringes is a rare event
The SSP does have staff assigned to assist PWID to access substance use treatment and to assist HIV seropositive PWID to access ART. The staff can make initial intake appointments for PWID at both substance use and ART programs, but do not have the capability to track persons who fail to show for their intake appointments
SSP staff regularly but informally interview program participants about whether the SSP is meeting their needs of sterile syringes and changes in drug use patterns in the community. SSP outreach workers also informally interview PWID in the community about access to sterile syringes and about changes in the patterns of drug use
Conclusion: The SSP is functioning very well
Cost-saving calculation
If the SSP budget is $500,000 per year, then the minimum number of new HIV infections that would need to be prevented is $500,000/$229,899 = 2.2, which rounds up to 3
Conclusion: The minimum cost-savings threshold would be averting 3 additional new infections per year
Is the SSP cost saving to society?
This question can be rephrased as: Given that there is some ongoing transmission of HIV in the community, if we reduced the supply of sterile syringes by 500,000 per year in a PWID population between 3000 and 7000, would we expect to see more than 3 additional HIV infections per year in the local PWID population?
All epidemiologic models that we are aware of would answer the question with a definite: Yes, reducing the supply of sterile syringes by this amount would definitely lead to more than 3 new HIV infections per year
Common experience with SSPs and HIV transmission in PWID populations would also indicate that such a large reduction in the supply of sterile syringes would generate more than 3 additional incident cases of HIV infection per year in the PWID population

Conclusion are underlined to separate sections of table

Given the high cost of treating HIV infections and the modest budgets of SSPs, it is extremely likely at any SSP functioning very well in an area in which HIV transmission is under control (HIV incidence of 1/100 PWID per year) will be preventing a sufficient number of new HIV infections to be cost saving to society.

As noted in the discussion of cost-effectiveness studies above, estimating the numbers of new HIV infections in the absence of an effective SSP requires extensive data and modeling of the likely HIV transmission dynamics in the local PWID population. Based on studies of recent HIV outbreaks among PWID, there are two likely scenarios if HIV transmission is not under control in the local PWID population [24, 25].

1. A temporary lapse in SSP services, an outbreak is detected, and the effectiveness of the SSP services are rapidly re-instated. This tends to produce modest outbreaks, with tens of excess new HIV infections among PWID.
2. An initial lack of SSP services or a long-term disruption in SSP services leading to a large HIV outbreak with hundreds of excess HIV infections.

Either of these scenarios is likely to generate high costs, in the millions of dollars.

Tables 2 and 3 provide details on recent HIV outbreaks in North American, Europe, and the USA [24, 25]. Athens, Greece, Bucharest, Romania, and Scott County, IN, USA, are examples of large HIV outbreaks; the other sites can be considered modest outbreaks.

**Table 2:** International outbreak details

International outbreaks					
Location	Outbreak year	Pre-outbreak case rates	Peak outbreak rate	Excess cases	Precipitating conditions
Athens, Greece	2011	10–20 HIV cases/year	525 cases over a 1-year period	505–515 cases	Economic recession; homelessness; low HIV prevention services
Bucharest, Romania	2011	5–12 HIV cases/year	308 cases/year	296–303 cases	Poverty; increase in synthetic drug use
Dublin, Ireland	2014	10–20 HIV cases/year	57 cases over 2-year period	37–47 cases	Economic recession; homelessness; increase in daily “snow blow” injections
Tel Aviv, Israel	2012	~ 40 HIV cases/year	73 cases over 1-year period	~ 33 cases	Homelessness; increase in synthetic cathinone use
Luxembourg	2013	< 4 HIV cases/year	68 cases over 4-year period	64 cases	Economic precariousness; homelessness; increase in cocaine use and decrease in heroin supply
Glasgow, Scotland	2015	~ 10 HIV cases/year	48 cases over a 1-year period	38 cases	Austerity; homelessness; increase in cocaine injecting
Southeastern Saskatchewan, Canada	2016	< 1 HIV case/year	16 cases over 2-year period	15 cases	Poverty; homelessness; increase hydromorphone use

**Table 3:** US-based outbreak details

US based outbreaks					
Location	Outbreak year	Pre-outbreak case rates	Peak outbreak rate	Excess cases	Precipitating conditions
Cabell County, West Virginia	2019	~ 2 HIV cases/year	82 cases over a 1-year period	80 cases	Low HIV prevention services, lack of access to HIV testing
Lowell, Massachusetts	2016	~ 0 HIV cases/year	5 cases over a 1-year period	5 cases	Homelessness, fentanyl injection, low HIV prevention services
Northern Kentucky/Hamilton County, Ohio	2017–2018	< 20 HIV case/year	157 cases over a 2 month period	137 cases	Increase in injection drug use starting in 2017
Philadelphia, Pennsylvania	2018	~ 33 HIV cases/year	71 cases over a 1-year period	38 cases	Incarceration, increase fentanyl injection
Portland, Oregon	2019	~ 12 HIV cases/year	42 cases over a 1-year period	30 cases	Methamphetamine injection
Seattle, Washington	2018	~ 17 HIV cases/year	52 cases over a 1-year period	35 cases	Homelessness; heroin and methamphetamine injection in combination; sex exchange among females
Scott County Indiana, USA	2014	< 1 HIV cases/year	227 cases over 3-year period	226 cases	Low employment rate; prescription oxymorphone; no HIV prevention services

## Discussion

Despite the vast scientific literature supporting the effectiveness of SSPs in reducing HIV transmission among PWID, there is often great stigma (often from community members and other groups) associated with injecting drug use and SSPs. Evidence from many studies note community opposition for SSPs, fearing that they may lead to increased drug use and crime [26]. It is not likely that a fully developed cost-effectiveness analysis of a local SSP or the more limited cost-savings analysis proposed here would change the attitudes of persons ideologically opposed to SSPs.

We would, however, like to suggest ways in which a positive cost-savings analysis could be used in support of SSPs in policy decisions.

First, a positive cost-savings analysis would provide an additional argument for supporters of SSPs. Supporters of SSPs already know that they are “doing the right thing” in terms of the ethics of “saving lives.” They also know that they are doing the right thing in terms of utilizing the science of HIV prevention. If the cost-savings analysis is positive, they would also be doing the right thing in terms of public finances. Given continuing attacks on SSPs in the USA, e.g., the closing of Scott County SSP [27], it is important that supporters have as multiple rationales for supporting SSPs.

Second, a positive local cost-savings analysis can be used to counter two “practical” arguments made by opponents of SSPs. A positive cost-savings analysis can be used to refute an argument that “We cannot afford to fund an SSP.” A positive local cost-savings analysis can also be used to refute the argument that “We are different from other areas, and the data from other areas does not apply to us,” as local data are used in the analysis.

Finally, a cost-savings analysis could be used to avoid placing burdensome restrictions on a local SSP. The cost-savings analysis applies to SSPs that are “functioning very well.” An overly restrictive SSP thus may not be cost-saving to the community.

## Limitations

Several limitations of this cost-savings analysis should be noted. First, it is not intended to convince persons who refuse to accept the data that SSPs can be effective in reducing HIV transmission or refuse to accept the data showing SSPs do not increase drug use. This analysis builds upon the large research base showing effectiveness of SSPs and lack of increases in drug use and will not be convincing to persons who reject those data. The cost-savings analyses are meant to show that, in addition to saving lives, SSPs are extremely likely to save money for their communities. Second, the analysis cannot be readily applied to SSPs that are not “functioning very well.” In particular, the cost-savings analysis cannot be applied to a local SSP that reaches very small numbers of PWID or that distributes very small numbers of sterile syringes. In those situations, the cost saving logic should be used to advocate for improving the operations of the local SSP.

The cost analysis presented here is based on conditions in the USA. Conditions in other countries, particularly low and middle income countries and in countries that do not have commitments to provide treatment for HIV seropositive persons, may be quite different, though the logic of the analysis would still apply.

## Conclusion

The procedures and analyses provided here were designed so that SSPs (1) in areas where HIV transmission among PWID is being controlled and (2) for SSPs that are functioning very well would be able to answer questions about their financial impact on the community with the statement noted in the introduction: “We have collected and analyzed the data on HIV transmission among PWID in our area and on the operations of our program, and, considering the high cost of treating HIV infections, we can be quite confident that our SSP is saving money for our community.” The analyses do utilize current knowledge of HIV transmission and of good practices for SSPs. The analyses are also relatively simple and can be conducted with data that should be available in most localities in the USA.

## Data Availability

Data sharing is not applicable to this article as no datasets were generated during the current study. The article contains a description of how the methods may be applied to local data.

## Abbreviations

ART: Antiretroviral therapy
CDC: Centers for Disease Control and Prevention
PWID: Persons who inject drugs
SSP: Syringe service program
UNAIDS: Joint United Nations Programme on HIV/AIDS
